# Editorial: Treatment for advanced breast cancer with brain metastases

**DOI:** 10.3389/fonc.2023.1268702

**Published:** 2023-09-12

**Authors:** Min Yan, Jin Yang

**Affiliations:** ^1^ Henan Breast Cancer Center, The Affiliated Cancer Hospital of Zhengzhou University & Henan Cancer Hospital, Zhengzhou, China; ^2^ Cancer Center, The First Affiliated Hospital of Xi’an Jiaotong University, Xi’an, China

**Keywords:** tyrosine kinase inhibitor, monoclonal antibody, antibody-drug conjugate, breast cancer, brain metastases

With the great revolution of anti-cancer therapy, the prognosis of patients with advanced breast cancer (especially for human epidermal growth factor 2 [HER2]-positive subtype) has been largely improved over decades. More and more patients with brain metastases appear during the long disease course. Brain metastases complicate breast cancer treatment and impact the overall prognosis. In addition to local therapy (radiotherapy or surgery), systemic therapy is becoming attractive in the treatment of patients with metastatic breast cancer and brain metastases. This Research Topic focused on novel agents, new insights into the known regimens, biomarkers for efficacy, and mechanisms of drug resistance in advanced breast cancer with brain metastases. Overall, four articles were collected in this Research Topic.

With the emergence of novel drugs, more options are provided for the treatment of brain metastases. Chen et al. published a review to feature currently available systemic therapy strategies and to outline novel drugs and ongoing clinical trials that may be available in the future. Previously studied systemic therapies for patients with HER2-positive advanced breast cancer and brain metastases included monoclonal antibodies (trastuzumab and pertuzumab), tyrosine kinase inhibitors (TKIs; lapatinib, neratinib, pyrotinib, and tucatinib), and antibody-drug conjugates (ADCs; trastuzumab emtansine and trastuzumab deruxtecan). For patients with hormone receptor-positive advanced breast cancer and brain metastases, treatments included endocrine therapy (tamoxifen), cyclin-dependent kinases 4/6 inhibitor (abemaciclib), and mammalian target of rapamycin (mTOR) inhibitor (everolimus). For patients with triple-negative advanced breast cancer and brain metastases, treatments included chemotherapy, bevacizumab, and poly (ADP-ribose) polymerase inhibitor (talazoparib).

Based on previous literatures, Huo et al. performed a meta-analysis of randomized controlled trials and single-arm clinical studies to analyze the currently most beneficial treatment for patients with HER2-positive breast cancer brain metastases. In randomized controlled trials, trastuzumab deruxtecan significantly improved progression-free survival and overall survival and was superior to other drug regimens. In single-arm studies, the objective response rate was more pronounced for trastuzumab deruxtecan (73.33%) and pyrotinib plus capecitabine (74.58%) regimens. The main adverse events of ADC were nausea and fatigue, while the main AE of small-molecule TKIs and monoclonal antibodies was diarrhea.

Wu et al. performed a bibliometric analysis to provide an updated summary on the development, hotspots, and research trends of brain metastases from breast cancer. A total of 693 researchers from 3623 institutions across 74 countries and regions published 2790 papers in 607 journals. There was a noticeable increase in publications in 2006. The United States was the dominant country with the most publications, followed by China. University Texas MD Anderson Cancer Center was the most productive institution, while Dana Farber Cancer Institution was the most cited. *Journal of Neuro-Oncology* published the most papers, while *Journal of Clinical Oncology* ranked first based on cocited analysis. Nancy U. Lin was the most productive and cited author with high influence. There was a focus on basic researches, clinical trials, local therapy, treatment optimization, and epidemiological studies regarding brain metastases from breast cancer. References focusing on pathogenesis, prevention, treatment, and prognosis were cited most frequently, among which the clinical trials of novel treatment attracted most attention from researchers. Reference citation burst detection suggested that new therapies such as novel TKIs and ADCs may lead the research trends in the future.

Regarding biomarkers for efficacy, Freeman et al. examined the association between Karnofsky performance status (KPS) and brain-specific progression-free survival in patients with breast cancer brain metastases treated with surgery and/or radiotherapy. Of 331 patients treated with surgery and/or radiotherapy who had available KPS data, 102 (31%) had KPS ≤60. In a multivariable analysis, KPS ≤60 was the only independent factors of brain-specific progression-free survival (hazard ratio, 1.86; 95% confidence interval, 1.20-2.88).

From the abovementioned literatures, we have seen the advance of drug therapy in HER2-positive breast cancer with brain metastases, anti-HER2 ADC trastuzumab deruxtecan and novel TKI pyrotinib have good intracranial and extracranial efficacy. For patients who do not need local therapy immediately at diagnosis of breast cancer brain metastases, such systemic therapies may be used preferentially to prolong the survival of these patients. We hope that the articles published in this Research Topic can help researchers enhance their understanding of treatments and prognostic factors for patients with advanced breast cancer and brain metastases.

## Author contributions

MY: Writing – original draft, Writing – review & editing. JY: Writing – review & editing.

